# Localizing Binding Sites on Bioconjugated Hydrogen‐Bonded Organic Semiconductors at the Nanoscale

**DOI:** 10.1002/cphc.201901064

**Published:** 2020-02-07

**Authors:** Melanie Koehler, Dominik Farka, Cigdem Yumusak, Niyazi Serdar Sariciftci, Peter Hinterdorfer

**Affiliations:** ^1^ Institute of Biophysics Johannes Kepler University Linz 4020 Linz Austria; ^2^ Linz Institute for Organic Solar Cells (LIOS), Physical Chemistry Johannes Kepler University Linz 4040 Linz Austria; ^3^ Institute of Solid State Physics Johannes Kepler University Linz 4040 Linz Austria; ^4^ Louvain Institute of Biomolecular Science and Technology (LIBST) Université Catholique de Louvain 1348 Louvain-la-Neuve Belgium

**Keywords:** epindolidione, hydrogen-bonds, organic semiconductors, quinacridone, TREC-AFM

## Abstract

Hydrogen‐bonded organic semiconductors are extraordinarily stable organic solids forming stable, large crystallites with the ability to preserve favorable electrical properties upon bioconjugation. Lately, tremendous efforts have been made to use these bioconjugated semiconductors as platforms for stable multifunctional bioelectronics devices, yet the detailed characterization of bio‐active binding sites (orientation, density, etc.) at the nanoscale has not been achieved yet. The presented work investigates the bioconjugation of epindolidione and quinacridone, two representative semiconductors, with respect to their exposed amine‐functionalities. Relying on the biotin‐avidin lock‐and‐key system and applying the atomic force microscopy (AFM) derivative topography and recognition (TREC) imaging, we used activated biotin to flag crystal‐faces with exposed amine functional groups. Contrary to previous studies, biotin bonds were found to be stable towards removal by autolysis. The resolution strength and clear recognition capability makes TREC‐AFM a valuable tool in the investigation of bio‐conjugated, hydrogen‐bonded semiconductors.

## Graphical abstract

**Atomic force microscopy** topography and recognition imaging is a powerful tool for the label‐free quantification of biotinylated substrates by localizing specific molecular‐recognition events at the nanoscale while simultaneously studying the surface morphology.

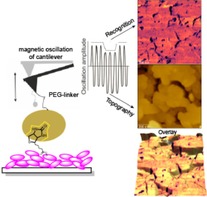

## 1 Introduction

In the past years, the rise of organic^1^, ^2^, ^3^ and biointegrated electronics^4^, ^5^, ^6^, ^7^, ^8^, ^9^ resulted in a big demand for advanced and multifunctional materials uniting a number of characteristics: low toxicity,^10^, ^11^ biocompatibility,^12^, ^13^, ^14^ low elastic modulus, stability,^15^, ^16^, ^17^, ^18^ and, ideally, low‐cost and abundance.^19^, ^20^ Many of these properties are met by the class of hydrogen‐bonded organic semiconductors (HB‐OSC′s). Their applications range from sensing and actuation, drug delivery, to ionic/ electronic signal transduction, which makes the perfectly suited to achieve specific interfacing between the bioelectronics and the targeted biosystem.^1^, ^2^, ^21^, ^22^

**Figure 1:**
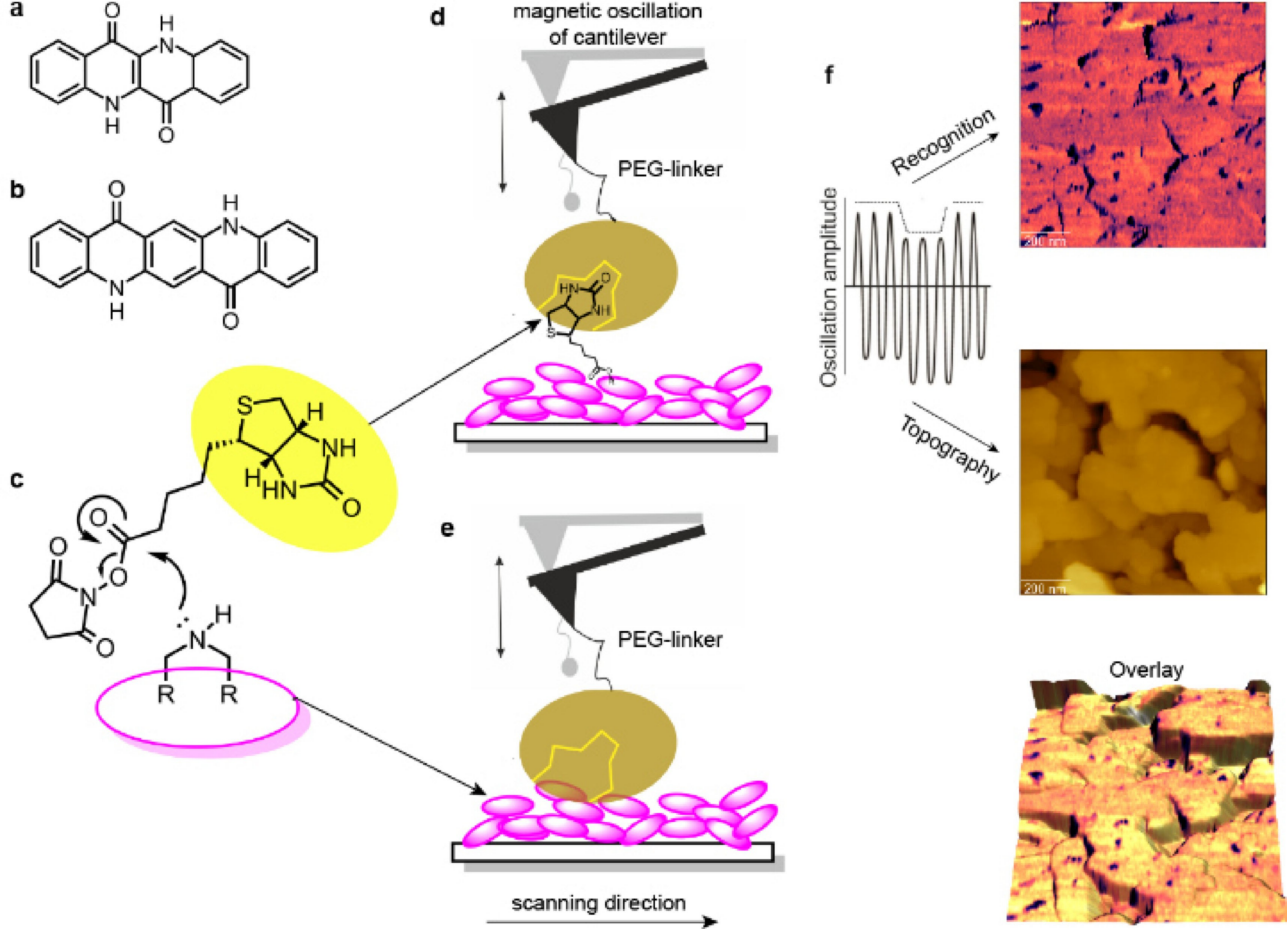
Experimental Setting. (a, b) Chemical structure of (a) Epi and (b) Quin. (c) Reaction mechanism of bio‐functionalization by biotin (yellow circle). (d–f) Specific (d) and unspecific (e) biotin detection on the HB‐OSC crystal based on topography and recognition (TREC) imaging with an avidin‐functionalized tip (brown circle). (f) Separation into two amplitude signals, resulting in topography (lower oscillation peaks) and recognition (upper oscillation peaks) information from a single surface scan at high resolution. Single‐targeted biotin molecules are visible as dark spots in the recognition image. The overlay of both images assigns location of binding sites to topographical features.

Here, we investigate two sets of model systems, the HB‐OSC′s epindolidione (Epi, Figure 1a) and quinacridone (Quin, Figure 1b) equipped with the lock‐and‐key system of biotin and avidin. Epi and Quin are readily available, as they are used as pigments in inkjet and photograph printers. Moreover, they have been previously shown to exhibit semiconducting properties^23^ with the ability for biocompatibility,^24^ as well as bioconjugation^25^, ^26^ (Figure 1c). The bioconjugation of both HB‐OSC's was previously investigated in the context of nature's most prominent lock‐and‐key system, biotin (vitamin B7, further B7) and (strept‐)avidin, one of the strongest known non‐covalent interaction.^27^, ^28^ While specific bioconjugation was shown with B7 acting as an anchor, their distinction from non‐specific, non‐covalent interactions remained challenging up to date. A robust and reliable analysis technique preserving functionality and capable to distinguish between specific and non‐specific binding phenomena is thus highly demanded. Since bioconjugates can be fabricated with a variety of biomaterials and interfaces, this requirement is not only important for the example presented here. A whole portfolio of well‐developed techniques has been applied to validate biomolecular attachment and to characterize nanomaterial structures (reviewed in^29^), including separation techniques (*e. g*. high‐performance liquid chromatography,^30^, ^31^ and field flow fractionation^32^, ^33^) and scattering methods (*e. g*. dynamic light scattering,^34^, ^35^ and fluorescence correlation spectroscopy^36^, ^37^). Fluorescence‐based techniques such as fluorescence spectroscopy or scanning confocal microscopy provide information concerning successful bioconjugation, conformational states of attached biomolecules, and stability of bioconjugated material.^38^, ^39^ Yet they require fluorescence labelling and suffer from the diffraction limit. Electron microscopy can only be operated in vacuum and requires extensive sample preparation, including exquisite steps such as freeze‐drying and staining or metal coating.^40^, ^41^ In contrast atomic force microscopy (AFM) can be carried out in aqueous and physiological environments without laborious sample preparation, staining or labelling, yielding a spatial resolution in the (sub)nanometer regime. This makes it an optimal tool to retrieve morphological information of bioconjugated surfaces and to image interactions between individual particles and biomolecules. (reviewed in^29^, ^42^, ^43^) AFM topography imaging has been applied to characterize the bioconjugation on a variety of nanomaterial – biomolecule platforms, including quantum dots, colloidal nanoparticles and carbon nanotubes associated to DNA„^44^, ^45^ as well as many other materials functionalized with different proteins.^45^, ^46^, ^47^, ^48^, ^49^ Besides topographical imaging, the capability of functionalizing AFM tips with biomolecules acting as ligands, makes it an outstanding tool for studying specific biomolecular recognition at the single‐molecule level and localizing receptor sites at the nanoscale. (reviewed in^50^)

Consequently, in the presented work, we set out to investigate the interactions between (biotinylated) Epi/ Quin and avidin via topography and recognition (TREC)‐atomic force microscopy (AFM).^51^, ^52^, ^53^ While TREC‐AFM has been reported for many biological applications,^50^ it has never been used for studying the specific oriented bioconjugation of organic nanomaterials or semiconductors. This mode enables a fast, stable and reliable mapping of specific biomolecular recognition events on bioconjugated surfaces. To overcome the limitations of speed and resolution, the avidin‐functionalized AFM cantilever is oscillated in a sinusoidal motion at small amplitudes (∼11–13 nm) close to its resonance frequency. The AFM tip senses the surface during lateral scans to simultaneously record topography images and localize specific binding sites between the avidin on the tip and the biotinylated substrate with nanometer accuracy and with less than 5 min scanning time. Amplitude oscillation changes arising from tip‐surface interactions are depicted and split into lower and upper parts. From these signals, topographical and recognition images are constructed, respectively.^54^ When the tip‐coupled avidin binds to the substrate exposed biotin molecules, only the upper parts of the oscillating amplitude are reduced as a result of specific recognition. Thus, upper movements of the cantilever are attenuated when an interaction between avidin and biotin takes place, because the AFM tip is temporarily bridged to the surface (Figure 1d–f). These interactions were localized and presented as dark spots in the recognition image (Figure 1f, upper panel) in correlation to the sample topography (Figure 1f, middle panel).

Using this method, we correlated binding events to topological features of the surface, the orientation of the molecules within their crystals, and related our results to specific binding sites presented by B7 covalently bound to the surface. After careful analysis of the data, we characterized bioactive binding sites in terms of their orientation, density, surface appearance, reproducibility and reliability with respect to different substrate preparation procedures, *e. g*. Epi and Quin evaporated onto substrates at different temperatures, which makes it possible to tune the crystal with respect to different sizes and orientations according to one's needs.

## 2 Results and Discussion

As previously reported,^27^ B7 conjugated to Epi and Quin is highly susceptible to hydrolysis, while (strept‐)avidin exhibited non‐specific binding to both surfaces. However, the question remains how avidin interacts with the surface and what mechanism leads to hydrolysis. Consequently, we prepared biotinylated samples of Epi and Quin evaporated to substrates at different temperatures and investigated their topology and affinity to avidin via TREC‐AFM. By utilizing this technique, the simultaneous observation of topography and surface‐tip interactions makes it possible to annotate avidin binding to specific surface features.

### 2.1 Epi‐Biotin Prepared at RT and 150 °C

**Figure 2:**
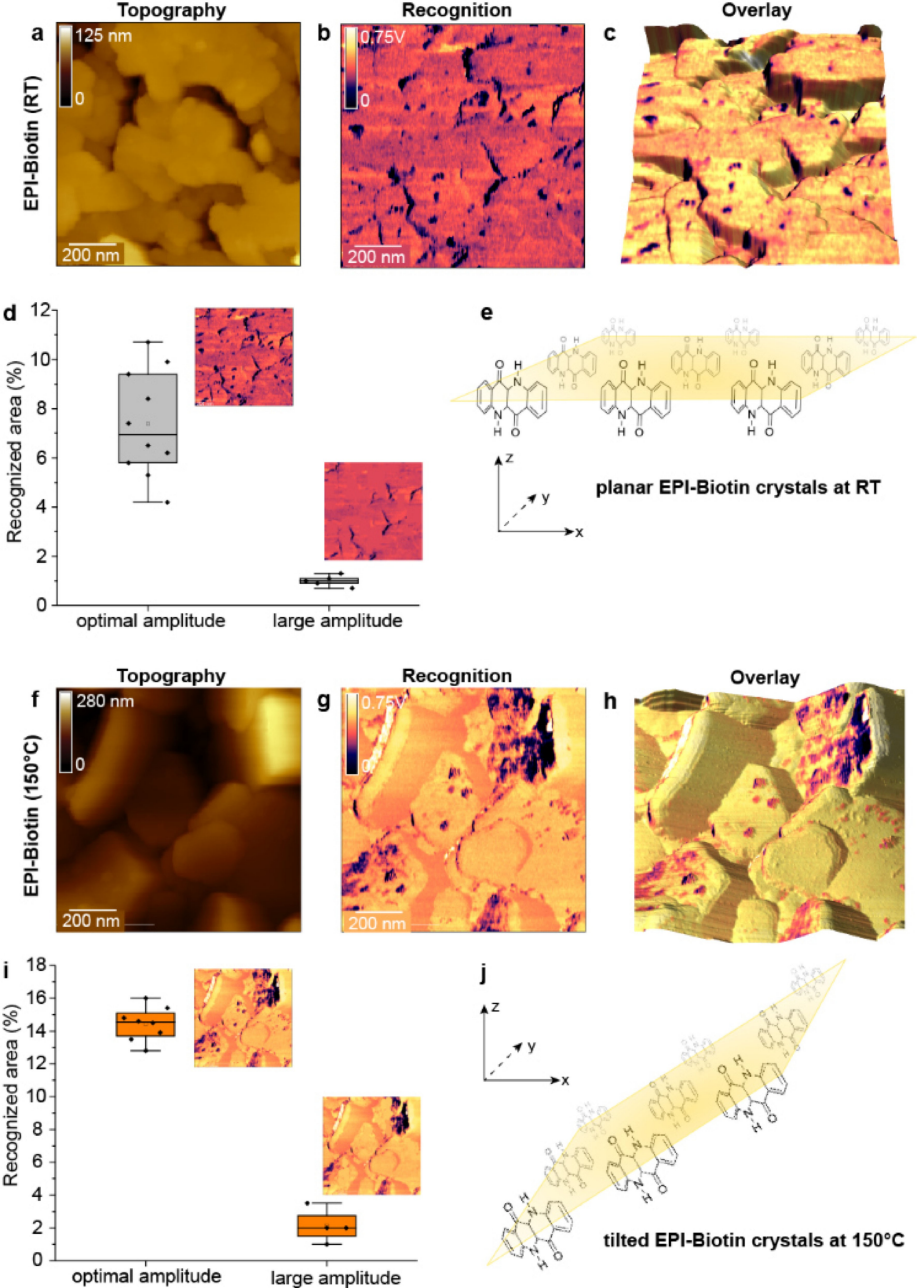
Biotin detection on Epi crystals (prepared at RT and 150 °C) based on TREC imaging. (a–c) Topography image (a), recognition image (b), and their overlay (c) of Epi surface area evaporated to a surface cooled to room temperature (RT). (f–h) Topography image (g), recognition image (g), and their overlay (h) of Epi surface area evaporated to a surface heated to 150 °C. Heating changed the relative orientation of the crystals from flat plateaus (e) to tilted planes (j). (d, i) Evaluation of recognition events on RT‐Epi (d) and 150 °C‐Epi (i) to distinguish specific binding at optimal amplitude. The overlay images (c, h) facilitate distinction of topologically caused non‐specific binding and specific recognition of biotin.

From Epi evaporated onto a sample and cooled to room temperature (RT), topography images were recorded as shown in Figure 2a. Crystals formed typical oblong shapes as previously reported,^27^ but quickly turned to spheroids of about 100 nm in size in water. This finding was rather surprising, but is an apparent consequence of the strong, hydrogen‐bond‐mediated interaction between material and solvent. Our observation contradicts previous findings,^27^ and appears to be a non‐permanent effect caused by swelling, due to biotinylation or exposure to water, rather than a permanent recrystallization. The recognition channel recorded with an avidin‐carrying AFM tip (Figure 2b) showed the presence of two characteristic signal sources: (i) specific binding where crystal boundaries meet and (ii) circular spots on top of the crystals. The first finding can be explained with the increased surface area at the crystal boundaries, leading to intrinsically increased surface interactions (Figure 2c), while the second observation can be attributed to specific interactions between B7 and avidin. The size of the spots with about 6.5±0.8 nm indicates that a single B7 surface molecule has recognized by the avidin on the AFM tip. These signals remained strong and measurable over several hours of measurement. Throughout our experiments (∼100 scanning hours), only one single event showed cleavage of the Epi‐B7 bond while interacting with avidin. This was observed as a sudden and definite disappearance of specific binding events after a single, strong signal was observed. Thus, we can rule out a significant effect of Epi‐B7 binding on the stability of biotinylation. The overlay (Figure 2c) and the presence of covalently bound B7 reveals a feature typically inaccessible via AFM: an indirect proof of molecular orientation within the crystal. It truly appears that the grain boundaries, despite being expected to be more reactive, do not show biotinylation. Our specific anchor, B7 can react only with Epi through forming an amide bond. Consequently, this means either, that grain boundary‐amines are less reactive (which is improbable), or that there is a preferential orientation of molecules within the grains, which we were able to detect via this technique. In this way, the grain boundaries are less reactive because of reactive amines being available strictly at the “top” or plateau‐surface. At the grain‐boundaries (the crystal sides) the avidin‐tag of our AFM‐tip does not reveal any specific interactions in hundreds of experiments. Consequently, the absence of B7 and further the absence of reactive amines can be concluded. An illustration of this molecular orientation is included in Figure 2e.

Epi evaporated onto heated substrates, larger crystals with 300–400 nm in diameter, formed as shown in Figure 2f. The oblong shapes origin from the crystals appearing tilted when compared to RT‐samples (Figure 2e, j) subsequently leading to a larger height difference. In addition, the results gained from the recognition channel changed dramatically compared to RT‐samples (Figure 2g). The interaction of the surface with the tip decreased. A significant decrease in non‐specific interactions may resulted from higher evaporation temperatures and a tilt in the crystals. The spot‐size of specific interactions was found to be 13.4±1.6 nm. The overlay of topology and recognition image (Figure 2h) confirmed the findings about the tilted crystal‐surfaces of the H‐bonded plane and the increased roughness. An illustration of that can be found in Figure 2j. These findings have a major impact for the application of Epi on sensors used in aqueous medium, as it indicates that multiple facets with different physical properties of the Epi crystal contribute significantly to the surface properties.

In order to statistically analyse our findings as well as to distinguish between specific and non‐specific interactions, we have quantified the recognized area of both Epi samples recorded at two different oscillation amplitudes (optimal and too large amplitude) (Figure 2d, i). Since setting the optimal oscillation amplitude of the cantilever is crucial for specific recognition, varying this parameter can be used as a quick and inherent specificity control without perturbation of the substrates.^55^ If well‐adjusted, the biotin‐avidin complex that forms between tip and surface exerts a non‐negligible force acting on the cantilever. Then the top peaks of the cantilever oscillations generate well‐defined recognition spots (Figure 2d) with sufficient background rejection. Under optimized conditions, the avidin covalently tethered to the tip and the target biotin conjugated to the substrate form a bond until the tip is laterally moved away from the target molecule to finally break the complex. In contrast, too large oscillation amplitudes lead to breaking of the avidin‐biotin complex at each oscillation cycle. In this case, no recognition spots are recorded, as shown in Figure 2d. After establishing the right amplitude, we performed several scans, followed by an increase of the free amplitude on the same position. In the RT‐Epi samples, we detected a clear difference between specific and non‐specific recognition (Figure 2d). Non‐specific interactions contributed to the overall area with only ∼1 %, whereas specific interactions were between 4–11 %. In the light of findings by Glowacki *et al*. to previous bioconjugation studies,^27^ this indicates that a large area of non‐specific bindings was caused by a relocation of streptavidin towards non‐specific binding sites. It also illustrates, that while fluorescent techniques provide a quick insight into bioconjugation efficiency, they do not provide a complete understanding of the underlying mechanisms. Thus, drawing conclusions with respect to specificity of binding based on results achieved solely from fluorescent techniques should be treated with care. On the contrary, in the 150 °C‐Epi samples (Figure 2i), the contribution of grain‐boundaries to non‐specific binding was minimal, whereas the strength of hydrogen bonds at the surface was the significant contribution. However, we could not decipher, whether these “activated hydrogen bonds” came from the solution, the crystal size and/ or evaporation parameters. The unspecific contribution amounted to an area of 2 % of the total area and the specific recognition, recorded at the previously established optimal amplitude, to 15 % of the total area. Again, the prevalent contribution was shown to originate from specific interactions.

### 2.2 Quin‐Biotin Prepared at RT and 150 °C

**Figure 3:**
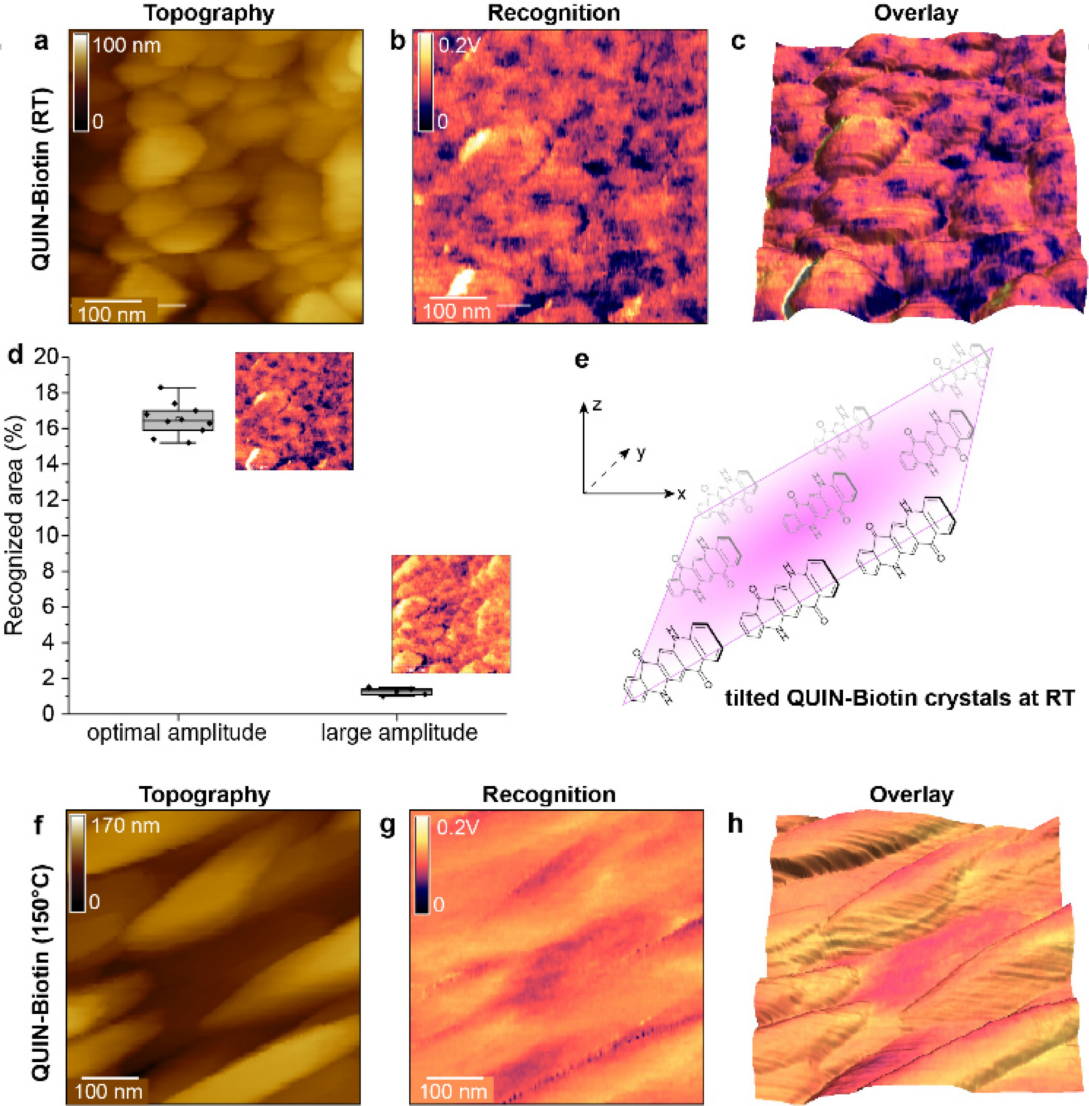
Biotin detection Quin crystals (prepared at RT and 150 °C) based on TREC imaging. (a–c) Topography image (a), recognition image (b), and their overlay (c) of Quin surface area evaporated to a surface cooled to room temperature (RT). (d) Evaluation of recognition events on RT‐Quin to distinguish specific binding at optimal amplitude. (f–h) Topography image (f), recognition image (g), and their overlay (h) of Quin surface area evaporated to a surface heated to 150 °C. Heating changed the relative orientation of the crystals from tilted planes (e) to elongated, cigar‐like structures with no clear orientation. The overlay images (c, h) facilitate distinction of topologically caused non‐specific binding and specific recognition of biotin.

To elucidate the effect of a slightly larger, but otherwise analogue molecular system on biotinylation and recrystallization in aqueous solution, we repeated our investigations using Quin (Figure 3). The topography of RT‐Quin revealed latent recrystallization of Quin into spherical/ egg‐like forms (Figure 3a), with the crystallites of ∼100 nm in diameter and thus highly similar to Epi. Only in the recognition channel, their difference is clearly visible (Figure 3b). First, a much smaller distribution of signal strength can be observed, paired with a larger area of recognized avidin‐biotin bonds. The overlay of both channels (Figure 3c) magnifies reveals that crevices between the round crystals contribute significantly to the recognition and that the reactive H‐bond faces are oriented towards the sides of the crystals.

The interpretation of the results gained with 150 °C‐Quin appears to be more complex (Figure 3f–h). The general trend of larger crystallites that forms at higher substrate temperatures can be confirmed as well as a tendency of crystals to retain the aspect ratio typical for this material, even in aqueous conditions; cigar‐forms of hundreds of nanometers in length were found (Figure 3f). Strikingly, the recognition channel (Figure 3g) did not reveal the clear presence of (single) B7, and only small areas of a possible interaction between avidin and the substrate were found. They mostly appear on lower crystal surfaces, which might indicate, that the top crystallites hinder recrystallization of the lower crystals, thus creating valleys with different physiochemical conditions, tens of nanometers wide and hundreds of nanometers long. Where we remain cautious about the Quin evaporated to hot surfaces, the RT‐samples were subjected to statistical evaluation and the specificity proof by varying the oscillation amplitude (Figure 3d). Again, the non‐specific recognition (revealed at high amplitude), contributes only to a small area of the whole scan being evenly distributed. The area contribution of recognition at optimal amplitude (*i. e*. specific interactions) ranges at 17 % and is less widely scattered than in Epi‐samples. It appears that the bigger spacing between the H‐bond‐forming functional groups has a positive effect on B7 binding. With the understanding of the binding sites gained from the experiments and supported by our statistical evaluation, we propose a possible orientation of Quin‐molecules in RT‐samples as shown in Figure 3e. The orientation of H‐bonds towards the sides implicitly implies a larger surface‐area for binding events and also appears to have a positive effect on the signal/noise ratio of in the recognition channel.

## 3 Conclusions

We have studied the HB‐OSC's Epi and Quin and their bioconjugation to B7 in aqueous conditions by applying high‐resolution TREC‐AFM with an avidin‐functionalized tip. Our study presents TREC‐AFM as a powerful tool for the label free quantification of biotinylated substrates (prepared under different conditions) by localizing molecular recognition events at the nanoscale in addition to simultaneously study the morphology.

We clearly demonstrated that specific interactions outweigh their non‐specific counterparts. The stability of HB‐OSC‐B7 bond was found to last over several hours in water. As previous studies were using a stream of water to remove superfluous B7,^27^ the combination of (i) the solvent, (ii) a higher content of dissolved, atmospheric gasses, and (iii) the additional mechanical and shear forces caused by the stream will likely play a significant role in hydrolysis. Consequently, auto‐hydrolysis must be reviewed as a mechanism of de‐biotinylation of surfaces.

In addition, we have shown that without statistical evaluation of the amplitudes observed through the TREC‐channel, non‐specific bindings can be easily misunderstood for the presence of B7. Strikingly, the reactivity of B7 also reveals the orientation of the molecules within the crystal and thus allows for a better understanding of the effect of processing conditions on active surface areas. Presenting TREC‐AFM on the well‐known lock‐and‐key system avidin – biotin demonstrates that this sophisticated methodology can be easily applied for studying and characterizing orientated bioconjugation of other organic nanomaterials or semiconductors at unprecedented resolution. This could strongly impact their use in biosensors – both with respect to novel applications as well as possible bottle‐necks.

## Experimental Section

### Materials

Epi was purchased from 1‐material Organic Nano Electronic (Quebec, Canada), synthetized and further purified as described elsewhere.^27^ Manufacturer and methods for Quin can be found in the same publication.^27^ N‐Succimidylbiotinate was obtained from Tokyo Chemical Industries (TCI) and used as received. Phosphate‐buffered saline (PBS) was obtained from Alfa Aesar (pH 7).

### Glass Silanization

All used glass slides were silanized with n‐octyltrichlorosilane (OTS) in order to avoid delamination of the pigments. The glass substrates and a volume of OTS was heated to 80 °C overnight in a sealed container. In our case, 60 μL of OTS in a ∼100 mL container was sufficient to achieve hydrophobic surfaces. Consequently, the samples were sonicated in toluene, isopropanol; then, we rinsed them in toluene, isopropanol and, and 18 MΩ water.

### Bioconjugation of HB‐OSC's

A 3 mM stock solution of N‐succinimidylbiotinate was prepared in dimethyl sulfoxide (DMSO) (used as received from Thermo Scientific) and stored in a refrigerator. For working solutions, a 20‐fold dilution in PBS was prepared, yielding a concentration of 0.15 mM. The substrate of choice (Epi or Quin) was fully covered in the working solution and left to incubate overnight in a sealed contained with high humidity. Then, the solution was removed and the substrate rinsed in a stream of 18 MΩ water.

### Bioconjugation of AFM Tips

Commercial magnetically coated cantilevers (MAC levers, Keysight) were functionalized with avidin (Sigma Aldrich) by a well‐established three‐step procedure: (i) amino‐functionalization of the cantilevers by gas phase silanization with (3‐aminopropyl)‐triethoxysilane (APTES) to convert the tip surface into a chemically addressable surface, [40] (ii) attachment of a distensible heterobifunctional polyethylene glycol crosslinker (acetal‐PEG_27_‐NHS), and (iii) coupling of avidin to the free end of the PEG chain via free amino groups.^56^, ^57^ The APTES coating was performed exactly according to Ebner et al.^58^ In brief, the cantilevers were washed with chloroform (3×5 min) and dried in a gentle nitrogen gas stream immediately before further treatment. Next, a desiccator (5 L) was flooded with argon gas to remove air and moisture. Then two small plastic trays (e. g. the lids of Eppendorf reaction vials) were placed inside the desiccator. 30 μL of APTES and 10 μL of triethylamine were separately pipetted into two trays and the AFM tips were placed close to the trays on a clean, inert surface (Teflon). The desiccator was closed and flooded with argon for one minute. After 2 h of incubation APTES and triethylamine were removed and the desiccator was again flooded with argon for 10 min. The tips were left inside for at least 2 days in order to cure the APTES coating. For coupling of the acetal‐PEG_27_‐NHS crosslinker, the APTES‐functionalized AFM tips were incubated in 0.5 ml of a 1 mg/ml solution of acetal‐PEG_27_‐NHS in chloroform containing 30 μl of triethylamine (TEA) as catalyst for 2 h. Subsequently, the tips were washed in chloroform (3×10 min) and dried in a gentle stream of nitrogen. Immediately before ligand coupling, the acetal group was deprotected by incubation of the acetal‐PEG_27_‐NHS functionalized tips in 1 % citric acid (in water) solution for 10 minutes, followed by rinsing in water (3×5 min) and drying as described above. For linking the avidin to the free end of the PEG chain, one portion of avidin solution (100 μL at ∼1 μM concentration) was pipetted onto the tips placed on Parafilm (Bemis NA) in a small plastic dish. A freshly prepared solution of sodium cyanoborohydride (NaCNBH_3_) (2 μL at ∼6% wt. in 0.1 M NaOH(aq)) was gently mixed into the avidin solution, and the cantilever chips were gently positioned with the cantilevers extending into the avidin drop for 1 h. Then, 5 μL of 1 M ethanolamine solution (pH 8) was gently mixed into the drop and incubated for 10 min in order to passivate unreached aldehyde groups. Finally, the tips were washed 3 times in PBS, and stored in individual wells of a multiwell dish containing 2 mL PBS per well until used in AFM experiments.

### AFM Topography and Recognition Imaging

Topography and recognition images were carried out using a commercial Keysight 5500 AFM (Keysight Technologies, Santa Rosa, USA) with a PicoTREC unit in PBS buffer. For TREC imaging in liquid, magnetically coated and avidin functionalized AFM cantilevers (type VII MAC lever, Keysight Technologies) with a nominal spring constant of 0.1 N/m in magnetic AC mode (Magnetic Alternating Current, MAC mode) and a quality factor (Q) of ∼1 in liquid were used. The driving frequency of the cantilever was 9–11 kHz with 11–13 nm amplitude. Set‐point was set to ∼20 % amplitude reduction during imaging in PBS. The lateral scan speed for all topographical and simultaneously acquired TREC images was 1 line/s at 256×256 pixels. Image size was varied between 0.5 and 2 μm and all images were taken using a closed loop scanner. Before starting the TREC measurements, the exact oscillation amplitude was determined using MAC mode amplitude‐distance cycles. The recognition spots for each TREC measurement were identified by a custom image analysing method, in which the background signal and the recognition signal in the recognition images were separated from each other by using threshold analysis.
